# Multiple displacement amplification for complex mixtures of DNA fragments

**DOI:** 10.1186/1471-2164-9-415

**Published:** 2008-09-15

**Authors:** Muhammad Shoaib, Sonia Baconnais, Undine Mechold, Eric Le Cam, Marc Lipinski, Vasily Ogryzko

**Affiliations:** 1 Université Paris-Sud 11, CNRS UMR 8126 Interactions Moléculaires et Cancer, Institut de Cancérologie Gustave-Roussy, 94805 Villejuif Cedex, France; 2 Institut Pasteur, URA 2171, Unité de Génétique des Génomes Bactériens, 75724 Paris Cedex 15, France

## Abstract

**Background:**

A fundamental requirement for genomic studies is the availability of genetic material of good quality and quantity. The desired quantity and quality are often hard to obtain when target DNA is composed of complex mixtures of relatively short DNA fragments. Here, we sought to develop a method to representatively amplify such complex mixtures by converting them to long linear and circular concatamers, from minute amounts of starting material, followed by phi29-based multiple displacement amplification.

**Results:**

We report here proportional amplification of DNA fragments that were first converted into concatamers starting from DNA amounts as low as 1 pg. Religations at low concentration (< 1 ng/*μ*L) preferentially lead to fragment self-circularization, which are then amplified independently, and result in non-uniform amplification. To circumvent this problem, an additional (stuffer) DNA was added during religation (religation concentration > 10 ng/*μ*L), which helped in the formation of long concatamers and hence resulted in uniform amplification. To confirm its usefulness in research, DP1 bound chromatin was isolated through ChIP and presence of DHFR promoter was detected using q-PCR and compared with an irrelevant GAPDH promoter. The results clearly indicated that when ChIP material was religated in presence of stuffer DNA (improved MDA), it allowed to recover the original pattern, while standard MDA and MDA without stuffer DNA failed to do so.

**Conclusion:**

We believe that this method allows for generation of abundant amounts of good quality genetic material from a complex mixture of short DNA fragments, which can be further used in high throughput genetic analysis.

## Background

**Figure 1:**
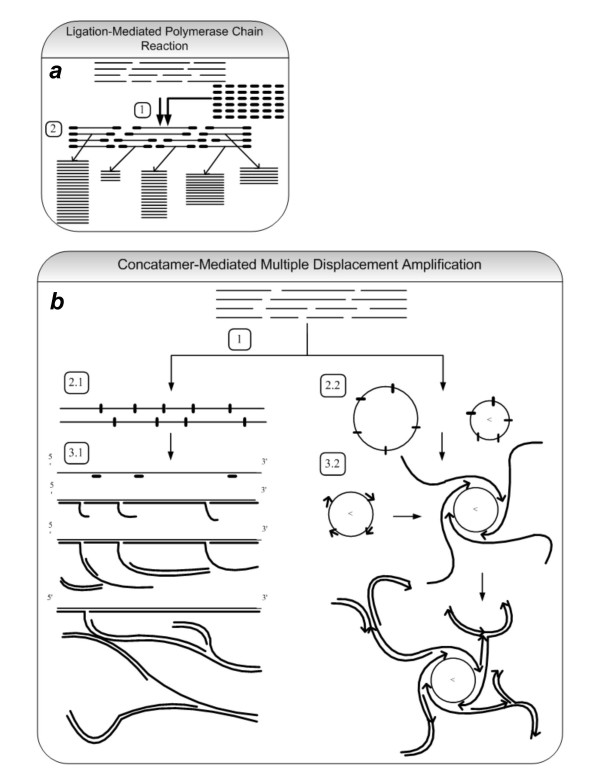
**Ligation-Mediated PCR and Concatamer-Mediated Multiple Displacement Amplification**. (a) The principle of Ligation Mediated PCR (LM-PCR). 1-Ligation with excess of primers, 2-Polymerase chain reaction of individual fragments. In LM-PCR, each fragment is amplified independently so that due to intrinsic differences among individual fragments, some fragments are amplified less efficiently than others. This results in non-uniform representation of original genetic material in the resultant amplicon, which consequently leads to loss of genetic information and inaccurate results. (b) The principle of concatamer-mediated multiple displacement amplification. 1-Religation of DNA fragments with T4 DNA ligase, which leads to two types of products, 2.1-Linear Concatamers and 2.2-Circular Concatamers. 3.1 and 3.2-Annealing of random hexamer primers and addition of phi29-DNA polymerase leads to concatamers-mediated multiple displacement amplification from linear and circular concatamers respectively.

Amplification of a complex mixture of relatively short DNA fragments, derived from mini scale experiments in chromatin immunoprecipitation (ChIP), degraded forensic material, cDNA synthesis, clinical diagnostics, stored tumor or other tissue samples is an area of genome research which has received scant attention. Abundant amounts and good quality of amplified material are required for high throughput analysis of this kind of complex genetic material. However, the currently available amplification techniques for complex mixtures of DNA fragments, often based upon PCR [e.g. Ligation-mediated PCR (LM-PCR)][1], do not amplify all fragments in equal proportion. In the later, every fragment is amplified independently and hence depending upon the presence or absence of GC-rich sequences and secondary (hairpin) structures in some DNA fragments, different elements of the mixture tend to amplify at different rates. This non-uniform amplification leads to relative loss of genetic material and ultimately can result in severe mis-representation of the fragments, producing inaccurate results and loss of important genetic information (fig. 1a).

Here, we focused on developing a methodology, which permits uniform amplification of a complex mixture of relatively short DNA fragments using small amounts of starting material. The principal novelty in the technique was in the ligation of small DNA fragments leading to the formation of long linear and circular DNA concatamers. As each individual small DNA fragment becomes part of a single large molecule, the intrinsic differences among individual DNA fragments, for example GC content and presence of secondary structures, should be averaged out (fig. 1b). Amplifying these concatamers using the phi29-DNA polymerase based Multiple Displacement Amplification (MDA) [2,3] should result in proportional amplification of the genetic material regardless of intrinsic differences among individual initial fragments.

We present here how the process of generating long circular &/or linear concatamers starting from very small amounts of a complex mixture of relatively short DNA fragments was optimized, ultimately to produce adequate quantity and quality DNA to be used for high-throughput genomic analysis.

## Results

### Multiple Displacement Amplification of Concatamers Allows Preservation of Initial Ratios of Various Fragments in a Complex Mixture

**Figure 2:**
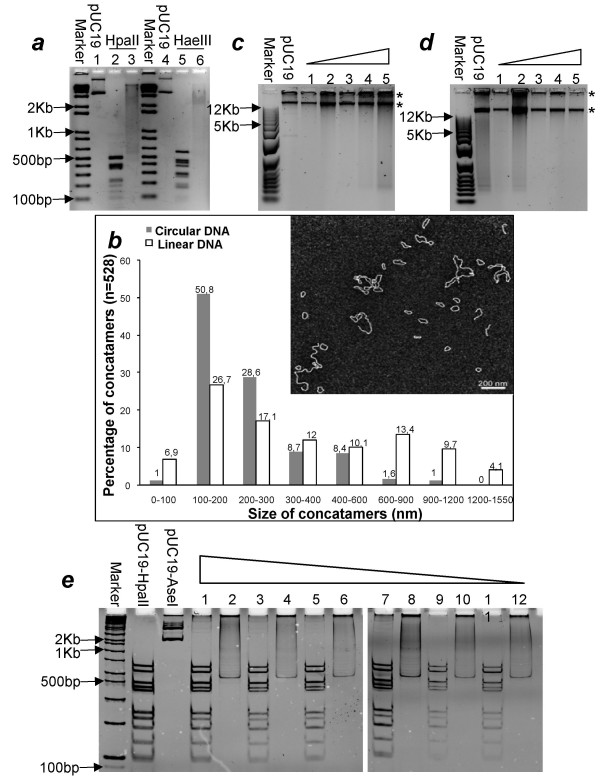
**Multiple displacement amplification of concatamers**. (a) Digestions and religations of 50 ng pUC19 with HpaII and HaeIII. Lanes 1,2 & 3 correspond to 50 ng each of pUC19 undigested, digested with HpaII, digested with HpaII and religated, respectively; while lanes 4,5 and 6 correspond to the same amounts of pUC19 undigested, digested with HaeIII, digested with HaeIII and religated, respectively. 2% agarose gel stained with EB was used for electrophoresis. (b) Frequency distribution of circular and linear concatamers. The horizontal axis shows the increasing size of concatamers in nanometers while the vertical axis shows the percentages of both linear and circular concatamers with respect to their size in nanometers. An example of electron micrograph showing the presence of both circular and linear concatamers as shown in the insert. (c) & (d) MDA of HpaII and HaeIII digested and religated samples respectively. Five dilutions (in ascending order, 1:1 pg, 2:10 pg, 3:100 pg, 4:1 ng, 5:10 ng) were made after religation in both cases along with untreated pUC19 (1 ng) taken as positive control. The amplification products are indicated by asterisks. 0.8% agarose gels stained with SYBR Green were used for electrophoretic analyses. (e) Re-digestions of HpaII digested, religated and amplified samples. Odd numbers represent the samples redigested with the same enzyme (HpaII) while even numbers represent samples redigested with a different enzyme (AseI). (1,2:1 pg, 3,4:10 pg, 5,6:100 pg, 7,8:1 ng, 9,10:10 ng). 10% polyacryamide gels stained with EB were used.

In the first set of experiments, we tested whether formation of concatamers, before isothermal amplification, of small amounts of starting material allows preservation of initial composition of the complex mixture of DNA fragments. We used pUC19 DNA digested with different restriction enzymes as a source of DNA fragments for religation and amplification. The digestion of pUC19 by almost any enzyme produces well recognizable and reproducible pattern, which can be compared later with that of the amplified DNA. Accordingly, pUC19 DNA was first digested either with HpaII (producing cohesive ends) or HaeIII (producing blunt ends), and the resulting fragments subjected to religation with T4 DNA ligase. Religations were done at relatively high DNA concentration (50 ng/*μ*L) to ensure the formation of linear and circular concatamers, which are a prerequisite for optimal multiple displacement amplification (MDA). As seen in fig 2a, HpaII and HaeIII digests yields distinct patterns of fragments ranging from 500 and 800 bp, respectively, to as low as 75 bp (lanes 2 & 5). The religation reaction converted these distinct patterns to long smears ranging from 0.6–12 Kb (lanes 3 & 6), representing long linear and circular concatamers. In most of our experiments, HpaII fragments gave longer religation products compared with those relegated from HaeIII products. This was expected, as blunt ends, generated by HaeIII, are ligated less efficiently.

To provide independent evidence that our religation procedure leads to generation of long linear and circular templates for phi29-based multiple displacement amplification (MDA), we subjected small aliquots of the religated samples to electron microscopy analysis. Fig. 2b exhibits an example of the electron micrograph obtained after analysis of HpaII digested and religated sample. The graph shows the length distribution of linear and circular concatamers. In total, we observed 41% of linear molecules and 59% of circles. More than 75% of the circles were between 100 nm (300 bp) and 300 nm (900 bp) in length [1 bp corresponds to 0.34 nm, [4]]. Compared to the average length of restriction digests obtained with HpaII (206 bp), this size is on average around 3 times longer and clearly indicates that most of the circles are results of religation of several fragments. Similar analysis with HaeIII sample showed 45.5% of linear and 54.5% of circular molecules, with nearly 80% of the circles between 100 nm (300 bp) and 300 nm (900 bp) in length, indicating approximately 3–5 fragments per circle (data not shown).

Fig. 2c &2d show results of MDA of the digested and religated DNA. Various dilutions were made after religation (10 ng/*μ*L, 1 ng/*μ*L, 100 pg/*μ*L, 10 pg/*μ*L, and 1 pg/*μ*L) which invariably gave 5–10 *μ*g of amplified material (> 1000 fold amplification). When the amplified product was run on 1% agarose gel typically two bands appeared: the upper band that remained in the well comprises some of the amplified material, while the lower band migrated in the gel. The appearance of distinct bands in some of the amplified lanes might be due to the formation of very large sized molecules after a rolling circle amplification of circular DNA tmeplates, which are beyond the resolution limits of agarose gel. The amplified material was also electrophoresed using 0.6% agarose gels but always obersved two bands (data not shown). The fact that some of the material does not enter the gel might be explained by extremely large size of the resulting amplification products and/or by the association of the amplified DNA with proteins. We favor the first explanation as: 1. In many cases, we could detect an increased viscosity of the reaction mixture after amplification reaction, suggesting the appearance of extremely long DNA strands. 2. After redigestion with restriction enzymes, we often observed that majority of DNA is cleared from the wells. 3. We have noticed that the upper band does not migrate even after treatment of amplified samples with a strong detergent e.g. SDS (data not shown). Our preliminary conclusion from these sets of experiments was that we could amplify concatamerized DNA starting from as low as 1 pg/*μ*L of DNA.

To see how uniformly the initial DNA fragments were amplified, we subjected the amplified products to redigestion using one of the restriction enzymes that was earlier used to generate a mixture of DNA fragments i.e. HpaII. A distinct pattern was obtained that was identical to the original digest pattern of HpaII, suggesting that the amplified product was indeed the result of uniform amplification of the original DNA templates (fig. 2e).

It seemed possible, however that not every pUC19 molecule was digested by HpaII before religation and amplification. Even if not detectable on lanes 2 & 5 (fig. 2a), minute amounts of undigested pUC19 could serve as an excellent template for MDA and thus give redigested products that would have the same pattern as the original pUC19 HpaII digest. To rule out this possibility, we used a different enzyme (AseI) to digest the amplification products. The rationale behind this control experiment is that in the case of amplification from true religated sample of HpaII digest, the order of restriction sites for AseI, present in the original HpaII digested, religated and amplified pUC19 DNA will be lost and thus leading to a smear after AseI digestion. In contrast, amplification from undigested pUC19 molecule should preserve the original AseI restriction pattern. As seen on fig. 2e (odd lanes), redigestion of amplified material using a different enzyme (AseI) produced a smeared product. This indicated that for most of the amplified material, the original order of fragments was not preserved in the amplified products, thus ruling out the possibility that the main template serving for amplification was undigested pUC19 DNA.

### Religation at Low Concentration Results in Loss of Proportionality among DNA Fragments

**Figure 3:**
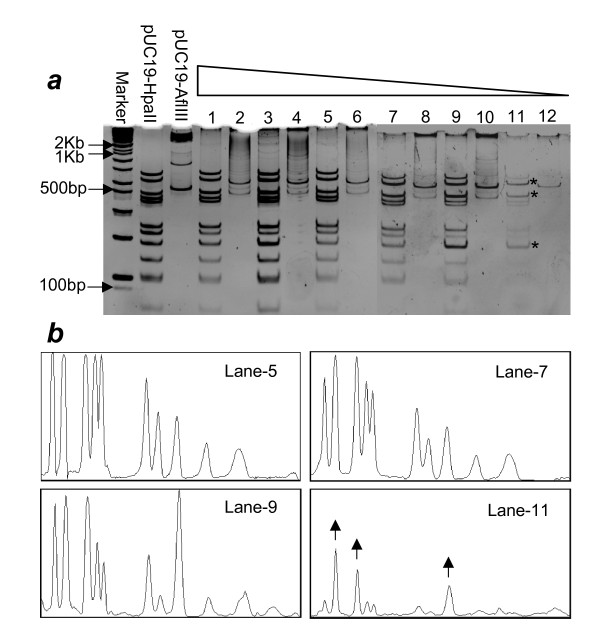
**Religation at low concentration lead to non-proportional amplification**. (a) Re-digestions of HpaII digested, religated and amplified samples. Religations were done at different concentrations. The concentrations go down in descending order from number 1 to 12. Six different concentrations were chosen i.e. 12 ng/*μ*L, 10 ng/*μ*L, 8 ng/*μ*L, 4 ng/*μ*L, 1 ng/*μ*L, 100 pg/*μ*L. Odd numbers represent the amplified samples redigested with the same enzyme (HpaII) while even numbers represent same samples redigested with a different enzyme (AflIII). (1,2: 12 ng/*μ*L, 3,4: 10 ng/*μ*L, 5,6: 8 ng/*μ*L, 7,8: 4 ng/*μ*L, 9,10: 1 ng/*μ*L, 11,12: 100 pg/*μ*L). The asterisks represent the fragments that are over amplified as compared to others. 10% polyacrylamide gels stained with EB were used for redigested samples. (b) Quantitative scanning of relative band intensities. The arrows shown in the scan of lane-11, corresponding to the asterisks in fig. 3a, represent DNA fragments that are relatively better amplified as compared to others in the same lane.

In the above experiments, we performed religation at relatively high DNA concentration (50 ng/*μ*L) and then made several dilutions before MDA. However, in real-life experiments, it is expected that the starting amount of DNA will be much lower. In the next set of experiments, religations were carried out at different concentrations starting from 12 ng/*μ*L down to 100 pg/*μ*L. As shown in fig. 3a, redigestions of the amplified products with the same enzyme (HpaII) preserved the pattern, while a different enzyme (AflIII) produced the expected smeared product. However, going down in the religated DNA concentration, the pattern of HpaII redigestion started to deviate from the original one. Although, we used equal amounts of amplified material for redigestion and loaded equal amounts of redigested DNA in all lanes, it was observed that at certain DNA concentration, the amplification yield starts to decrease, generating less amplified material and hence observed low signal in the last lanes in fig. 3. We also observed that some fragments were completely lost while some were only diminished especially in lane-11. The asterisks and arrows in fig. 3a &3b respectively, represent the fragments that are relatively better amplified than others. The selective loss of most of the fragments in this experiment could have been due to self-circularization of these fragments, which, consequently amplify as individual molecules, instead of amplifying as part of concatamers. This leads to a significant bias in the relative efficiencies of the various DNA fragments during amplification. The relative intensities of various fragments were quantitatively measured using image processing software 'Image J' [5]. The scans shown in fig. 3b clearly indicates that as we go down in the DNA concentration during religation, we get non-uniform amplification evident by the aberrant peaks in the scans of lanes 9 & 11.

### Use of Stuffer DNA

In order to circumvent the problem of self-circularization, we then decided to use 'stuffer DNA' – an excess of heterologous DNA, digested with a restriction enzyme and added to the 'target' DNA before religation. The purpose of stuffer DNA addition was to standardize the ratio of DNA amounts to the amounts of enzymes, and to ensure formation of concatamers instead of self-circularized fragments in case of very small amounts of starting material. We considered that it was preferable to use a plasmid DNA as a stuffer. The advantage of a plasmid is that it affords two easy controls for the quality of amplification product, as explained below. Theoretically, two problems can arise: 1. Insufficient concentration of DNA in religation mixture leads to self-circularization; 2. Amplification product could result from undigested stuffer DNA molecules, which serve as an excellent template for MDA. Both potential problems can be addressed by control digestion of the amplified product with two different restriction enzymes. First restriction enzyme should be the same that was used for the generation of stuffer DNA. A second restriction enzyme should be chosen to produce a restriction digest pattern, which is different from that of the first one. The digest with the first enzyme should give a pattern identical to the digest of original plasmid, serving as a positive indication that there was no self-circularization, which would otherwise lead to uneven amplification. The digest with the second enzyme helps to address the problem of under digestion, as the preservation of the pattern in this case would serve as a warning that the amplification product originated mostly from the undigested stuffer DNA molecules.

**Figure 4:**
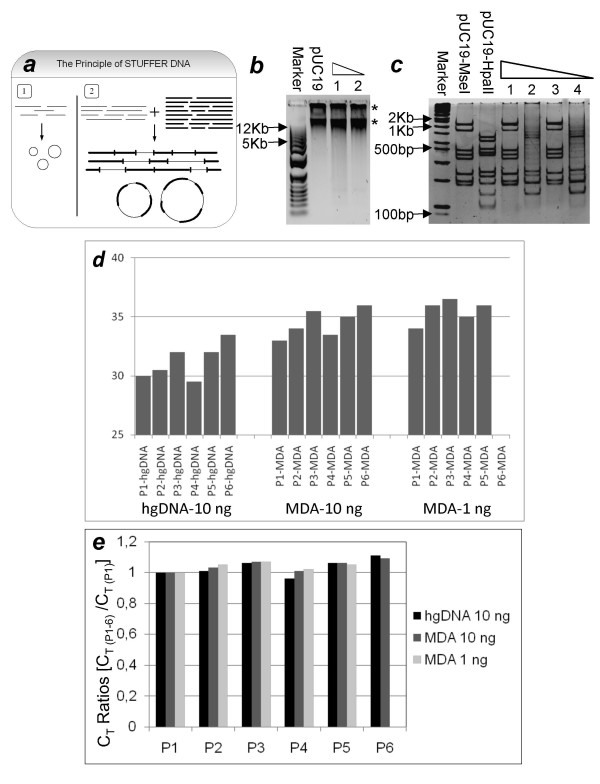
**Application of human genomic DNA with pUC19 as Stuffer DNA**. (a) The schematics of the idea of Stuffer DNA. 1-In case of much diluted samples, relegation preferentially leads to self-circularization of fragments, which are then amplified as individual molecules. 2-The addition of Stuffer DNA derived from an unrelated source to the sample DNA favors intermolecular ligation and leads to the formation of long concatamers, both linear and circular. These long concatamers can then be reliably amplified using MDA technique. (b) Amplification of hgDNA with stuffer DNA using MDA technique. Two different amounts of hgDNA were taken (1:10 ng/*μ*L and 2:1 ng/*μ*L) and a fixed amount of pUC19 (100 ng) was used as stuffer DNA. The amplification products are indicated by asterisks. 0.8% agarose gel stained with SYBR Green was used for electrophoresis. (c) Re-digestions of samples [previously digested with AseI and MseI (both gives identical cohesive ends), religated and amplified], with the same enzyme (MseI) and a different enzyme (HpaII). Odd numbers represent the samples redigested with MseI, while even numbers represent HpaII digests (1,2: 10 ng and 3,4: 1 ng). Asterisks indicate HpaII fragments that do not contain internal MseI sites and thus are preserved after MseI digestion and religation. 10% polyacryamide gel stained with EB was used for electrophoresis. (d) q-PCR data of 10 ng and 1 ng hgDNA samples MDA amplified with stuffer DNA. 6 different primer pairs were used for quantitative analysis. As a template for q-PCR, native hgDNA (left graph), and the MDA-amplified DNA (10 ng – middle graph, 1 ng – right graph) were used. Shown are C_*T *_values calculated for each fragment according to Materials and Methods. (e) To see if the amplification preserves the proportions among different genomic regions, for each MDA experiment, ratios were calculated between C_*T *_values of different fragments, taking P1-hgDNA as reference for hgDNA control samples and P1-MDA for MDA processed samples. Data for P6-MDA in case of 1ng input is not shown, as it did not give the correct amplification product.

As a model to test the usefulness of stuffer DNA, we used human genomic DNA (hgDNA) from HeLa cells (target DNA), while plasmid pUC19 was used as a stuffer DNA. hgDNA was digested with AseI, whereas pUC19 was digested with MseI. Both enzymes produce DNA fragments with compatible ends. Digestion of hgDNA with MseI gives very small fragments that are difficult to monitor with quantitative-PCR (q-PCR), we therefore used AseI instead. (The HpaII and HaeIII used in our preliminary experiments with pUC19 cannot be used for the analysis of genomic DNA, because because extensive methylation of human genomic DNA does not allow obtaining DNA fragments of sufficiently small size. Therefore, we had to choose different enzymes). After digestion of hgDNA, two dilutions were made, i.e. 10 ng/*μ*L & 1 ng/*μ*L. To each of these samples a fixed amount (100 ng) of pUC19, digested with MseI, was added, so that the final concentration of hgDNA + stuffer DNA became approximately 10 ng/*μ*L. This concentration was previously shown to be sufficient for avoiding self-circularization (fig. 3). After religation, the samples were subjected to MDA. Fig. 4b shows the amplification products after MDA and amplification yield was estimated to be approximately 1000 fold. To control the quality of the amplification product, aliquots from the final amplified products were redigested with the original restriction enzyme (MseI), and a different enzyme (HpaII). Redigestion with MseI produced pattern identical to the original one, while the digest with HpaII consistently gave a smear (fig. 4c), confirming that the amplification preserved the original composition of the mixture, and resulted mostly from true religation of the DNA fragments. The HpaII redigests also yielded some bands superposed on a smear (fig. 4c; lanes 2 & 4). These bands represent the HpaII sites present between the MseI sites, therefore they remain preserved despite the scrambling of fragments during religation.

In order to confirm that hgDNA was also amplified in a uniform way in this experiment, we subjected the amplification products to quantitative-PCR (q-PCR) analysis using different primer pairs, which amplify various regions of hgDNA. Six different hgDNA fragments (correspondingly six different primer pairs) were selected which amplify at different rates. Native hgDNA was taken as a reference. Fig. 4d, shows results corresponding to two hgDNA samples amplified employing MDA in the presence of stuffer DNA during religation. As one can see, the presence of every DNA region targeted by q-PCR analysis could be detected in the amplification products, although in smaller amounts as compared to the hgDNA control, indicated by shifted C_*T *_values shown in fig. 4d. This is explained by the dilution of the target DNA by the stuffer DNA present in the amplification products. Most importantly, however, the relative amounts of different DNA fragments targeted by q-PCR were very similar between the modified MDA-amplified products and the native hgDNA. We also calculated ratios among C_*T *_values of different fragments taking P1-hgDNA amplification fragment as a reference for hgDNA samples and P1-MDA amplification fragment for the two MDA samples correspondingly. As shown in fig. 4e, the ratios for MDA samples were conserved as compared to those of control hgDNA samples. (The deviation range is between 1–9%). Therefore, we conclude that the ratios between various DNA fragments were preserved in our amplification experiment.

### Application to Chromatin Immunoprecipitation

**Figure 5:**
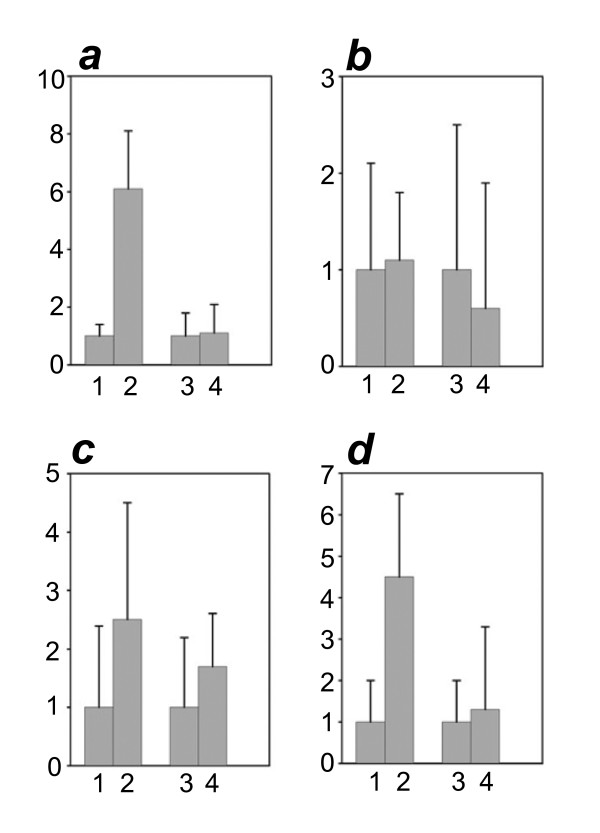
**Analysis of chromatin immunoprecipitation**. (a) DP1 complex with DHFR and GAPDH promoters. NIH 3T3 cells were transiently transfected with pBBHN.DP1 vector and subjected to the chromatin immunoprecipitation procedure. Shown is the ratio between amounts of DFHR (lanes 1 and 2) and GAPDH DNA (lanes 3 and 4) pulled down from the DP1 transfected sample (lanes 2 and 4) and GFP transfected sample (lanes 1 and 3), considered as a nonspecific background (mean of 2 experiments). 500 *μ*g of chromatin were used for the ChIP. 5% of the input chromatin for each sample was decrosslinked, processed and analyzed in the same way by q-PCR. For each sample the value of the signal is presented as a percent of input. (b) MDA without any religation. Immunoprecipitate from the same experiment as in fig. 5a was diluted 100 fold and subjected to MDA before q-PCR analysis. (c) MDA with religation. Immunoprecipitate from the same experiment as in fig. 5a was diluted 100 fold and then subjected to religation, MDA and q-PCR as above. (d) MDA with religation in the presence of stuffer DNA. Immunoprecipitate from the same experiment as in fig. 5a was diluted 100 fold and then subjected to religation in the presence of 100 ng of HaeIII digested pUC19 and then MDA and q-PCR as above.

To test whether our methodology was useful for a research application, we performed analysis of chromatin immunoprecipitation samples. We used binding of DHFR promoter with transcription factor DP1 as a model in our experiments. NIH3T3 cells were transiently transfected with a vector expressing in vivo biotinylated DP1, and the DP1 bound chromatin was isolated as described previously [6]. The presence of DHFR promoter was detected with q-PCR and compared with an irrelevant GAPDH promoter. Chromatin from NIH3T3 cells, transfected with GFP was used as a negative control. As seen on the fig. 5a, the DP1 bound chromatin contains significant amount of DHFR promoter, compared to the control chromatin immunoprecipitate, and no difference between the two samples is seen in the case of q-PCR analysis of GAPDH promoter. To test our improved MDA method, we diluted the samples 100 fold and performed three different amplifications: A. Direct MDA amplification without religation, B. With a sample subjected to religation before MDA, C. With a sample subjected to religation in the presence of stuffer DNA before MDA. The amplified material was analyzed by q-PCR for the presence of DHFR and GAPDH promoter containing fragments. As seen from the fig. 5b–d, MDA of the low amounts of the immunoprecipitated material does not allow to recover the original pattern presented on the fig. 5a, unless it was first religated in the presence of stuffer DNA. We conclude that the use of MDA for the uniform amplification of DNA mixtures obtained from the chromatin Immunoprecipitation experiments benefits from religation in the presence of stuffer DNA.

## Discussion

A fundamental requirement for genomic studies is the availability of DNA of adequate quantity and quality. Ample amounts of good quality DNA (at least 300–500 ng) are required for the systematic analysis of DNA acquired by mini scale experiments in chromatin immunoprecipitation (ChIP), stored tumor or other tissue samples, degraded forensic material, and cDNA generation, etc. [7-10]. Often, the desired quantity of DNA can be obtained by conventional PCR-based DNA amplification techniques e.g. LM-PCR [1,11] or balanced PCR [12,13]. However, the quality of amplified product is frequently hampered by the amplification bias introduced because the quantitative representation of various DNA sequences before and after PCR amplification is not uniform, especially if the DNA consists of complex mixtures of short fragments or it is modestly to highly degraded. To solve this problem, we modfied the existing MDA technique in such a way that the new methodology allows amplifying the sample DNA with uniform representation of different components of the starting material in the resultant product. In this protocol, the sample DNA that consists of a complex mixture of relatively short DNA fragments is religated to form linear and circular concatamers. These concatamers are then amplified using multiple displacement amplification (MDA). The resultant amplicon is expected to give an equal coverage of the original genetic material and therefore preserve better the composition of original DNA sample. A related method was previously proposed by Wang and colleagues [14], namely RCA-RCA (Restriction and Circularization Aided – Rolling Circle Amplification) to amplify formalin-fixed, paraffin-embedded DNA specimens, but it was primarily focused to address the shortcomings in whole genome amplification techniques most notably MDA. Although the authors described restriction digestion and religation procedure in their technique, it was not shown how the process of generating long linear and circular concatamers was controlled. In our methodology, we optimized the procedure and showed that during religation, going beyond certain minimum of DNA concentration favors self-circularization of individual fragments, which are poor candidates for MDA. Keeping in view the intrinsic differences among different DNA fragments, these self-circularized fragments will have an amplification disadvantage and will be under-represented in the resultant amplicon. Therefore, it is important to take into account the DNA concentration during religation. Moreover, in case of very low DNA concentration during religation (DNA fragments obtained from ChIP experiment), we have proposed another modification of the technique i.e. use of stuffer DNA, which is a DNA from unrelated source and is used to optimize the formation of long concatamers.

Phi29-based MDA is a highly reliable approach for generating abundant quantities and good quality amplified product from the template DNA without the need for thermal cycling and starting from very small quantities (picograms or even less) of sample DNA [2,15-18]. This amplification reaction relies on the excellent properties of the enzyme phi29-DNA polymerase [19-21], like strand displacement and processive synthesis at the rate of 25/50 nucleotides per second [22,23]. The polymerase has an inherent 3'-5' proofreading exonuclease activity [19,21,24]. It appears to have an accuracy comparable to other polymerases with a 3' proofreading exonuclease [21] which, is generally in the range of 10^-6 ^to 10^-8 ^[25], allowing high fidelity replication of input DNA template [21,18]. The use of random hexamer primers for the initiation of DNA synthesis in this technique circumvents the need for specific primers. However, a drawback is its requirement for a long linear or circular template. The short molecular templates will be decreasing in size after each amplification round, due to average position of the random primer somewhere along the molecule. Accordingly, MDA has largely been used for amplifying large circular DNA templates such as plasmids and bacteriophage in the form of rolling circle amplification (RCA) [3,17]. It has also been successfully employed in whole genome amplification (WGA), with an important advantage of exhibiting very little bias in amplification of different DNA sequences, and thus helping to preserve the original composition of amplified material. WGA is used to generate DNA in ample amounts to analyze Single Nucleotide Polymorphisms (SNP), restriction fragment length polymorphism (RFLP), and for comparative genome hybridization (CGH) [2,8,26]. Sequencing of DNA in case of clinical samples prior to PCR-based amplifications [8,27] and cell free cloning of single circular synthetic DNA molecules which cannot be cloned in E. Coli, has also been described using phi29-DNA polymerase, taking advantage of RCA and strand displacement properties of the enzyme [22]. Since, amplification of complex mixture of relatively short DNA fragments originating from a variety of sources has largely remained a bottleneck in high throughput genetic analysis, the formation of long linear or circular concatamers should alleviate this problem and thus significantly expand the application range for MDA.

In this project, we asked whether formation of long linear or circular concatamers could help to amplify a complex mixture of short DNA fragments. pUC19 plasmid DNA was used as a model because comparison of the digestion patterns before and after amplification can serve as a convenient way to monitor any bias in amplification, as it would lead to changes in relative abundance of different bands. The first question that we addressed was the minimal amounts of template that can be used for ligation and MDA. In the first set of experiments, we demonstrated that we could obtain sufficient amounts of amplified material starting from as little as 1 pg of DNA. The fact that it is the concatamerization that plays an essential role in our approach is based on several observations. First, the long amplification products observed in our reactions do not appear if we use digested DNA that was not religated. Secondly, redigestion of the amplification products with the same enzyme that was used to obtain the initial fragment mixture reconstitutes the original digestion pattern, while digestion with a different enzyme yields a smeared product, indicating that most of the template used for amplification is a true product of religation after digestion, and not coming from minute amounts of undigested pUC19. The third argument is our direct observation by electron microscopy of formation of concatamerized linear and circular molecules that are an excellent template for MDA.

It is known that the DNA concentration during the religation step is crucial in the formation of concatamers. In our experiments, religation at low DNA concentration led to self-circularization of the fragments, which were then amplified as single molecules. As a result, we observed a loss of some DNA fragments when the amplification product was redigested with same enzyme. The minimum DNA concentration that is required to avoid the self-circularization (10 ng/*μ*L) was still higher than the amounts typically obtained in mini scale experiments in ChIP, from various kinds of biopsies and forensic samples etc. To circumvent this problem, we used stuffer DNA. The addition of heterologous DNA to the sample ensured the formation of concatamers, and made the procedure more robust. In addition, the use of plasmid DNA as a stuffer serves as a good quality control for the amplification product, giving a good indication that the amplified mixture is amplified without a bias, before engaging in further expensive analytical procedures. Finally, we demonstrate here that MDA with stuffer DNA can be used for proportional amplification of DNA obtained from chromatin immunoprecipitation experiment, and hope that its usefulness for other research applications will be shown in further work. One of the potential drawbacks of using stuffer DNA is the dilution of the DNA of interest with heterologous DNA molecules. However, at relatively modest ratios between target and stuffer DNA, the presence of stuffer DNA can be tolerated and even prove beneficial. For example, when the amplified material is labeled for hybridization experiments, the presence of additional DNA fragments flanking the probe can enhance the hybridization signal, if measures are taken to suppress nonspecific hybridization. On the other hand, in the case of high ratios between target and stuffer DNA, a further development of the proposed procedure can be envisaged. Namely, stuffer DNA can be designed to contain sequences that can be used to selectively remove it from the amplification product after its redigestion with the enzyme that was used for its preparation. In this way, the target DNA can be enriched after amplification and the MDA be repeated, if necessary.

We believe that our approach to generation of abundant quantities of good quality amplified product will find many applications in genomic research. In our experience, a mixture of DNA fragments as small as 100–500 bp (e.g. obtained from ChIP experiments) at a concentration of 1–10 ng/*μ*L, ligated with 100 ng of stuffer DNA (preferably a commercial vector like pUC19, restriction digested with an enzyme producing blunt end DNA) at a final concentration of at least 10 ng/*μ*L, is sufficient as an input for 10 phi29-based MDA reactions, typically producing more than 1000 fold amplification. This amplified product, representing the composition of the initial DNA mixture, is suitable for further high throughput genomic analysis.

## Conclusion

The method presented in this paper is a modification of phi29-based MDA technique, allowing generation of abundant amounts of genetic material from mixtures of short DNA fragments and is tolerant to the intrinsic differences among DNA fragments. The technique uniformly amplifies the template DNA starting from very low amounts relying upon the formation of long linear and circular concatamers. The use of stuffer DNA during religation made the process even more robust by ensuring the formation of long concatamers and at the same time served as a good quality control for the amplified product. We expect that development of a protocol that representatively amplifies complex mixtures of DNA fragments will have a significant impact on the feasibility of high-throughput genomic analysis to unravel valuable genetic information from limiting DNA templates.

## Methods

### DNA and Primers

pUC19, a 2686 bp high copy number plasmid used in our experiment [28], was prepared by double cesium purification method. Human genomic DNA (hgDNA) was purified from HeLa cells using ChargeSwitch hgDNA Mini Tissue Kit (Invitrogen). For q-PCR analysis six different primer pairs were selected which amplify various regions of hgDNA stated as follows: Primer P1-TNFRSF11b/OPG gene (OPG-ChIP-F: TAGGGCCAATCAGACATTAGT, OPG-ChIP-R: GACGCAGTTGGAGTGTGTC), Primer P2-HERC5 gene, (HERC5-ChIP-F: GCCTGCCAAGTCACTCTCA, HERC5-ChIP-R: TCGGGGAACCGCAGCCTCA) Primer P3-WNT5A gene (WNT5aChIPex1F: CGGGGCGACTTTCACCTATT, WNT5aChIPex1R: AGGTGCCCCCAGTTCATTCA), Primer P4-part of the exon 2 of N-MYC protooncogene (gN-MYC 644F: GGCGTTCCTCCTCCAACAC, gN-MYC 737R: CGTTTGAGGATCAGCTCGC), Primer P5-mbActin 32–52 gene (mbActin gene 32–52-F: TGGCCGTCAGGCAGCTCA, mbActin gene 32–52-R: ACCGAGCGTGGCTACAGCTT), Primer P6-mbActin 33–53 gene (mbActin gene 33–53-F: AGGAAGAGGATGCGGCAGTG, mbActin gene 33–53-R: GCTTCACCACCACAGCTGAG). Primers P1–4 were kindly provided by Dr. David Cappellen (CNRS UMR 8126, Institut de Cancérologie Gustave-Roussy). Primers P5 & P6 were from Sigma-Genosys. For the q-PCR analysis of chromatin immunoprecipitated material, the following primers were used; DHFR 5': GCGGAGCCTTAGCTGCACAA, DHFR 3': TACCAGCCTTCACGCTAGGA, GAPDH 5': CCAATGTGTCCGTCGTGGATCT, GAPDH 3': GTTGAAGTCGCAGGAGACACC. The fragment amplified by these primers are P1–235 bp, P2–175 bp, P3–357 bp, P4–112 bp, P5–158 bp, P6–108 bp, DHFR-135 bp and GAPDH-190 bp long. All q-PCR reactions gave a unique product of expected size, as confirmed by 1% agarose gel analysis (data not shown).

### Restriction Digests and Ligations

HpaII and HaeIII enzymes were purchased from Sigma-Aldrich and New England Biolabs (NEB), correspondingly, both at a concentration of 10,000 U/mL. AseI and MseI were purchased from NEB at a concentration of 10,000 U/mL while AflIII, also from NEB has a concentration of 5000 U/mL. High concentration T4 DNA ligase (5000 U/mL) was supplied by QBIOgene. 1 *μ*g of pUC19 was usually digested under standard conditions. 100 ng of digested DNA was taken for religation and reactions were set up at 10 *μ*L. Ligations were done at 16°C overnight to obtain maximum ligation efficiency. All enzymes were inactivated by heat after completion of reactions. Quantitative determination of relative band intensities in fig. 3 was done using image processing software named 'Image J' version 1.38× [5].

### Transmission Electron Microscopy Analysis

5 *μ*L of the solution containing 0.5 *μ*g/ml of purified DNA, diluted in buffer (Tris-HCl 10 mM, pH 7,5 NaCl, 50 mM, EDTA 1 mM) were deposited onto a 600 mesh copper grid covered with a thin carbon film activated by a glow discharge in the presence of pentylamine as previously described [29]. Grids were washed with aqueous 2% uranyl acetate to contrast DNA using positive staining technique. DNA images were performed using Transmission Electron Microscope Zeiss 912AB at 120 kV in filtered dark field mode. DNA size was determined using 'Image J' software on 528 molecules of HpaII digested and relegated sample and 497 DNA molecules of HaeIII sample.

### MDA Reactions

Phi29-DNA polymerase was supplied by Epicentre Biotechnologies (10,000 U/mL). For our reactions, it was diluted in the dilution buffer supplied with the enzyme to a concentration of 5000 U/mL (600 U/mL final reaction concentration). 10× reaction buffer was prepared containing 370 mM Tris-HCl, 100 mM MgCl_2_, 500 mM KCl, 50 mM (NH_4_)_2_SO_4_, 2% Tween, 100× BSA (10 *μ*g/*μ*L) and H_2_O. From this 10× buffer, 2× buffer was prepared by adding 2 mM (final) dNTP's mix, 2 mM DTT, and H_2_O. Phi29-DNA polymerase was added to the 2× buffer and incubated at room temperature for 10 minutes. Immediately before setting up the reactions, random hexamer primers (50 *μ*M final concentration, supplied by NEB) were added. The final percentages of all the three ingredients in the final phi-29 mix were 63% 2× buffer, 12% phi29-DNA polymerase and 25% random hexamer primers. Reactions were set up in small volumes (600 nL per sample), which contains 200 nL of DNA template, and 400 nL of phi29-mix (2× buffer + phi29-DNA polymerase + random hexamer primers). Reactions were upscaled five times so as to make the volumes of samples workable, which were then diluted after amplification in ultra pure H_2_O (supplied by QBIOgene). Reactions were overlaid with 10 *μ*L of bio-technology grade mineral oil (Sigma-Aldrich) to prevent evaporation, then centrifuged briefly and checked visually to make sure the aqueous phase formed a small sphere at the bottom of the tube. Reactions were incubated in a thermocycler at 30°C for 6 hours, then heated at 70°C for enzyme inactivation and finally held at 4°C until analysis.

### DNA Analysis by Electrophoresis

Digestion and religation samples were analyzed on 2% agarose gel in TAE 1× buffer. For analyses of MDA samples, one sample from each reaction was taken out and separated on 0.8% agarose gels in TAE 1× buffer. Ready-Load 1 Kb plus DNA ladder (Invitrogen Life Technologies) was used. Ethidium Bromide (EB) was from Sigma. Where increased sensitivity was required, SYBR Gold was used as DNA staining dye (supplied by Invitrogen Life Technologies). The images were taken on Chemidoc Imager (BIO-RAD) and were analyzed using Quantity One software, (version 4.6, build 036). 10% polyacrylamide gel electrophoresis (PAGE) was used for redigestion of MDA processed DNA as it gives good resolution in case of comparing the patterns before and after amplification. Polyacrylamide gels were always stained with EB. Quantitative scanning of relative band intensities was done using software 'Image J' [5].

### Quantitative-PCR Analysis

hgDNA samples with stuffer DNA religated in two different concentrations, i.e. 10 ng/*μ*L and 1 ng/*μ*L, were subjected to q-PCR analysis. As stuffer DNA, 100 ng of pUC19 plasmid was added to the samples prior to religation. After two rounds of isothermal amplification, approximately 10 ng was taken from each sample to be used with each of the six primer pairs in q-PCR reactions. The final reaction volume was set at 25 *μ*L, where 12.5 *μ*L of SYBR Green PCR Master Mix (supplied by Applied Biosystems in 2× concentration) was used. 1 *μ*L (12.5 *μ*M) of each reverse and forward primers were added to the reaction and the rest was completed by DNA and *H*_2_*O*. The samples were run in 96-well plates in ABI-Prism 7000 Sequence Detection System (Applied Biosystems), and analyzed on 7000 System SDS Software v1.2. Quantitative analysis were performed using the standard curve method. The data used for quantification were verified by analysis of the dissociation curves and by running the amplified products on 1% agarose gels (data not shown). A standard amplification curve was generated for all the samples. This curve is a graphical representation of a function of number of PCR cycles and the amount of florescence generated. (X-axis indicates the cycle number when PCR started generating the florescence signals, while Y-axis shows the amount of florescence). We took *C*_*T *_[Cycle Threshold, which is the point at which amplification plot/curve crosses the threshold and is reported as the cycle number at this point [30]] value at midpoint of the slope of the amplification curve of each sample with all the primer pairs. We generated a graph comparing them simultaneously with the same midpoint value of hgDNA control with the respective primers. In order to demonstrate that initial proportions were preserved among the fragments after MDA, the *C*_*T *_values for the fragments with different primers were analyzed in such a way that ratios were calculated among *C*_*T *_values taking P1 amplification fragment as reference for hgDNA control samples and P1-MDA for MDA processed samples. *C*_*T *_values were taken from single experiments.

### Chromatin Immunoprecipitation

Chromatin immunoprecipitation from the transiently transfected NIH3T3 cells was performed as described earlier [6]. Briefly, cell were transfected with Polyfect (Qiagen), incubated in a biotin-containing medium and two days after treated with 1% of formaldehyde. Cells were pelleted by centrifugation and suspended in 1 mL of SDS buffer (50 mM Tris at pH 8.1, 0.5% SDS, 100 mM NaCl, 5 mM EDTA and protease inhibitors) and incubated for 10 minutes on ice. Cells were then pelleted by centrifugation, resuspended in 400 *μ*L of IP buffer (0.3% SDS, 1.1% Triton ×100, 1.2 mM EDTA, 16.7 mM Tris at pH 8.1, 167 mM NaCl, and protease inhibitors), and disrupted by sonication, yielding genomic DNA fragments of a size of 100 to 500 bp. For each immunoprecipitation, 50 *μ*g of chromatin was diluted to a final volume of 300 *μ*L in IP buffer. Chromatin was pre-cleared for 1 hour by addition of 40 *μ*L of blocked protein-A beads (Pierce, cat. 20334) (50% slurry protein-A-agarose, 3 mg/mL BSA, 0,1 mg/mL salmon sperm DNA, in IP buffer). Samples were next incubated for 3 hours with 40 *μ*L of streptavidin coated magnetic particles (Promega, cat. Z5481). Beads were then washed twice with 2% SDS followed by 3 washes with LiCl wash buffer (100 mM Tris at pH 8.0, 500 mM LiCl, 1% NP40, 1% deoxycholic acid), or 5 washes with the LiCl wash buffer [normal wash [6]]. Samples were then decrosslinked by an overnight incubation at 67°C in 60 *μ*L of 300 mM NaCl solution. Proteinase K and 5× Proteinase K buffer (50 mM Tris at pH 7.5, 25 mM EDTA, 1.25% SDS) were then added to the samples. After two hour incubation at 45°C, DNA from the samples was purified using Qiagen miniprep columns (cat. 27106). Purified DNA was recovered in 50 *μ*L of water. In addition to the precipitated samples, 5% of the input chromatin for each sample was decrosslinked, processed and analyzed in the same way by q-PCR. For each sample the value of the signal was calculated as a percent of input.

## Authors' contributions

MS, SB and UM performed the experiments. VO, ML and ELC designed and coordinated the study. MS and VO drafted the manuscript. All authors read and approved the final manuscript.
